# Fulminant Amoebic Colitis

**DOI:** 10.4269/ajtmh.24-0430

**Published:** 2024-10-22

**Authors:** Juan Carlos Cataño, Valentina Montoya

**Affiliations:** ^1^ Infectious Diseases Section, University of Antioquia Medical School, Medellin, Colombia;; ^2^ Infectious Diseases Section, CES Clinic, Medellin, Colombia

**Figure 1.**
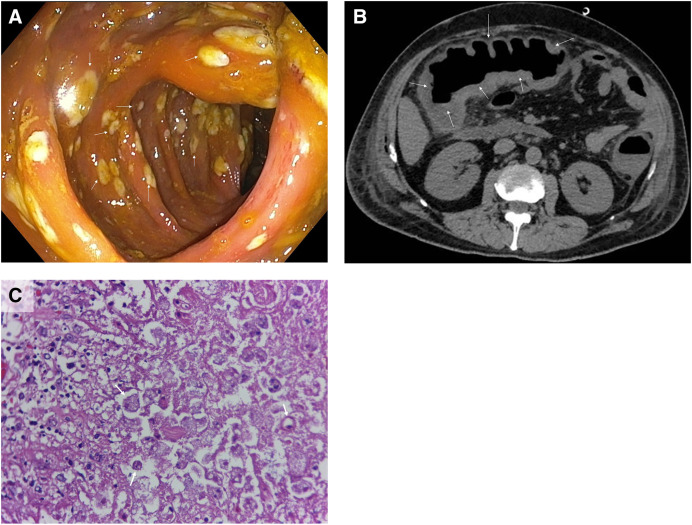
(**A**) Colonoscopy. Colon inside view showing an inflamed mucosa, with multiple amoebic ulcers (arrows). (**B**) Abdominal computed tomography scan showing diffuse thickening of the wall of the colon (arrows), associated with inflammatory changes in the pericolonic fat, suggesting severe colitis. (**C**) Colon histopathology with hematoxylin and eosin stain, showing extensive areas of transmural coagulative and liquefactive necrosis, with the presence of amoebic trophozoites (arrows) extensively invading the tissue.

A previously healthy 58-year-old man from Medellin, Colombia, presented to the emergency department with 1 day of malaise, subjective fever, severe abdominal pain, constipation, and rectal bleeding. On physical examination, he appeared sick, in acute pain, and dehydrated. Vital signs showed a blood pressure of 110/60 mm Hg, a pulse of 115/min, and a temperature of 37.8°C. There were no mouth lesions, and chest auscultation was normal, but on abdominal examination, the patient had slow peristalsis, hepatomegaly (20 cm total span), and pain, without clear signs of peritoneal irritation. Based on these symptoms, an initial abdominal computed tomography (CT) scan was performed, which showed severe circumferential thickening of the intestine walls and perirectal ganglia, resembling a neoplastic lesion; a colonoscopy then showed severely inflamed mucosa, with multiple ulcers, and abundant fibrinopurulent material in the rectum, cecum, sigmoid colon, and ileocecal valve (Figure 1A). The patient underwent a derivative colostomy, but after surgery, colostomy became necrotic, and a second abdominal CT scan showed diffuse thickening of the wall of the colon, associated with inflammatory changes in the pericolonic fat, suggesting severe colitis (Figure 1B). An exploratory laparotomy revealed complete necrosis from the lower rectum to the cecum, with areas of perforation, associated peritonitis, and no healthy colon tissue. As a result, the patient underwent a total colectomy and a terminal ileostomy. The colon histopathology showed extensive areas of transmural coagulative and liquefactive necrosis, with the presence of amoebic trophozoites causing extensive tissue invasion (Figure 1C). After surgery, the patient was treated with a combination of metronidazole and teclozan, resulting in clinical improvement.

Fulminant amoebic colitis is a rare disease with high morbidity and mortality. Sometimes it can have an aggressive clinical course, including colonic perforation, necrotizing colitis, and toxic megacolon, which often need urgent surgical treatment, and has a mortality rate of up to 40–50%.^1^^,^^2^ The diagnosis of invasive disease can be suspected by endoscopic findings that include multiple ulcers with exudates, usually involving the cecum, ascending colon, and rectum, but a confirming diagnosis requires the identification of parasite trophozoites in biopsy specimens.^3^^,^^4^ The therapeutic approach for fulminant colitis includes surgical resection of the compromised colon and antibiotic therapy.^5^
